# Comparison of the diagnostic efficacy between two PCR test kits for SARS‐CoV‐2 nucleic acid detection

**DOI:** 10.1002/jcla.23554

**Published:** 2020-09-25

**Authors:** Yu Lu, Limin Li, Shan Ren, Xin Liu, Lanzuo Zhang, Wei Li, Hongli Yu

**Affiliations:** ^1^ Department of Clinical Laboratory Liuzhou People's Hospital Liuzhou China

**Keywords:** Diagnostic efficacy, PCR kit, SARS‐CoV‐2

## Abstract

**Background:**

To compare the diagnostic efficacy between two different real‐time reverse transcription polymerase chain reaction (RT‐PCR) test kits for severe acute respiratory syndrome coronavirus 2 (SARS‐CoV‐2) nucleic acid detection and provide references for laboratories.

**Methods:**

Throat swab samples from 18 hospitalized patients were clinically diagnosed with coronavirus disease 2019 (COVID‐19) and 100 hospitalized patients without COVID‐19 were collected. SARS‐CoV‐2 nucleic acid was detected in throat swab samples with RT‐PCR test kits from Sansure Biotech Inc (Hunan, China) and Shanghai BioGerm Medical Biotechnology Co., Ltd.(Shanghai, China). The sensitivity, specificity, positive predictive value (PPV), negative predictive value (NPV), and kappa value were analyzed, and three parallel tests were performed with three weakly positive samples.

**Results:**

The sensitivity, specificity, PPV, NPV, and kappa value of the Sansure PCR kit were 0.833, 1.000, 1.000, 0.971, and 0.894, respectively, and the sensitivity, specificity, PPV, NPV, and kappa value of the BioGerm PCR kit were 0.944, 1.000, 1.000, 0.990, and 0.966, respectively. For the three parallel tests, the coefficient of variation value of the BioGerm PCR kit in all three samples was the smallest for both the ORF1ab and N gene.

**Conclusion:**

The detection efficacy of the BioGerm PCR kit for SARS‐CoV‐2 nucleic acid detection was relatively higher than that of the Sansure PCR kit.

## Graphical abstract

To compare the diagnostic efficacy between two different real‐time reverse transcription polymerase chain reaction (RT‐PCR) test kits for severe acute respiratory syndrome coronavirus 2 (SARS‐CoV‐2) nucleic acid detection and provide references for laboratories; Throat swab samples from 18 hospitalized patients were clinically diagnosed with coronavirus disease 2019 (COVID‐19) and 100 hospitalized patients without COVID‐19 were collected; SARS‐CoV‐2 nucleic acid was detected in throat swab samples with RT‐PCR test kits from Sansure Biotech Inc(Hunan, China) and Shanghai BioGerm Medical Biotechnology Co., Ltd. The sensitivity, specificity, positive predictive value (PPV), negative predictive value (NPV), and kappa value were analyzed, and three parallel tests were performed with three weakly positive samples.

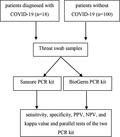

**Abbreviations**
CI: confidence interval
COVID‐19: coronavirus disease 2019
Ct: cycle threshold
CV: coefficient of variation
NPV: negative predictive value
ORF1ab: open reading frame 1ab
PPV: positive predictive value
RT‐PCR: Real‐time reverse transcription polymerase chain reaction
SARS‐CoV‐2: severe acute respiratory syndrome coronavirus 2

## 1 INTRODUCTION

In the final months of 2019, a cluster of pneumonia cases of unclear etiology was first noted in Wuhan, Hubei, China. The etiology of these pneumonia cases was soon identified as a new type of coronavirus.^1^ This virus was named severe acute respiratory syndrome coronavirus 2 (SARS‐CoV‐2), and the disease it causes is known as coronavirus disease 2019 (COVID‐19).^2^ Thus far, more than 84,000 COVID‐19 cases have been identified in China. Globally, approximately 5.2 million cases were reported as of May 23, 2020 (https://gisanddata.maps.arcgis.com/apps/opsdashboard/index.html#/bda7594740fd40299423467b48e9ecf6). The pandemic has created an enormous burden on health systems, as well as society and the global economy. Successful management of the spread of COVID‐19 depends on the timely and accurate diagnosis of patients with acute SARS‐CoV‐2 infection and accurate detection of asymptomatic carriers. However, the common clinical symptoms and laboratory examination findings of COVID‐19 are not unique.^3^ Real‐time reverse transcription polymerase chain reaction (RT‐PCR) is the most sensitive and specific assay and therefore has become the current standard diagnostic method for the diagnosis of COVID‐19.^4^, ^5^

According to Zhang et al,^6^ specimens such as nasal or throat swabs, sputum, lower respiratory tract secretions, peripheral blood, and feces from patients with COVID‐19 can be used to detect SARS‐CoV‐2 nucleic acid. A positive SARS‐CoV‐2 nucleic acid result from respiratory tract or blood samples is the basis of clinical diagnosis, and two consecutive negative nucleic acid test results are one of the standards for being discharged from the hospital.^7^ Thus far, many COVID‐19 RT‐PCR kits have become commercially available, and the majority of them use the open reading frame 1ab (ORF1ab) and the nucleocapsid protein as the major testing targets.^8^ As of May 23, 2020, the Foundation for Innovative New Diagnostics had listed 317 molecular assays on their website as being on the market (www.finddx.org/covid‐19/pipeline). However, independent assessment of these products is not yet publicly available. Maximization of the sensitivity and specificity of these test kits is critical to global efforts to control the spread of SARS‐CoV‐2.

To compare and analyze the detection performance of different SARS‐CoV‐2 nucleic acid detection kits, two kinds of domestic reagents were selected for parallel detection of a series of samples from Liuzhou People's Hospital in Guangxi, China, which is a designated hospital for patients with COVID‐19, to provide references for laboratories.

## 2 MATERIALS AND METHODS

### 2.1 Participants and sample collection

This study was approved by the Ethics Committee of Liuzhou People's Hospital. Patients with confirmed COVID‐19 infections who were admitted to Liuzhou People's Hospital from January 2020 to February 2020 were recruited. The patients were diagnosed according to National Health Committee guidance, and these diagnoses were further confirmed by RNA detection of SARS‐CoV‐2 in the Chinese Center for Disease Prevention and Control. A total of 18 patients, including 11 men and 7 women, with a mean age of 35.94 ± 16.32 years were enrolled, and throat swab samples were collected from them. For the control group, throat swab samples from 100 hospitalized patients without COVID‐19 (including 61 men and 39 women with a mean age of 36.50 ± 19.93 years) were collected during the same period.

### 2.2 Test kits and sample testing

Two virus nucleic acid RT‐PCR test kits from different companies were used: Sansure Biotech Inc (Hunan, China; Lot No. 2 020 007) and Shanghai BioGerm Medical Biotechnology Co., Ltd. (Lot No. 20200304A). Basic information on and the technique index of these two test kits are listed in Table 1.

**Table 1:** Basic information and technique index of Sansure and BioGerm test kits for SARS‐CoV‐2 nucleic acid

Test kits	Target genes	Nucleic acid extraction method	Nucleic acid volume (μL)	Amplification temperature	Number of Cycles	Analysis of Results		Minimum detection limit(copy/mL)	internal standard
						positive	negative		
Sansure	*ORF1ab*/N	magnetic bead method	20	60°C	45	S‐type amplification curve and Ct ≤ 40	Ct > 40	200	Yes
BioGerm	*ORF1ab*/N	magnetic bead method	5	55°C	40	S‐type amplification curve and Ct ≤ 35	Ct > 38	1000	No

### 2.3 Nucleic acid extraction

Nucleic acid was extracted from the samples using magnetic beads following the manufacturer's recommended protocol (Zhongyuan, Chongqing, China). Briefly, throat swab samples from both patients with COVID‐19 and patients without COVID‐19 were first inactivated with a water bath at 56°C for 30 minutes. Then, 200 μL of the inactivated sample was transferred to a 1.5‐mL reaction tube with working buffer (250 μL extraction buffer I + 250 μL extraction buffer II + 4 μL magnetic beads + 15 μL protease K) and heated at 55°C for 4 minutes. Samples were placed in the magnetic bead separator to remove the supernatant before extraction buffer III (600 μL) was added. Afterward, the supernatant was removed again in the magnetic bead separator, and 40 μL eluent was added to separate the extracted nucleic acid from the magnetic beads. Finally, the samples were placed in the magnetic bead separator for 3 minutes to remove the magnetic beads.

### 2.4 qRT‐PCR analysis

A volume of 20 and 5 μL nucleic acid that was extracted from patients with COVID‐19, and patients without COVID‐19 was subjected to analysis with the previously mentioned Sansure and BioGerm PCR kits, respectively. Amplification was performed using Applied Biosystems^™^ 7500 Real‐Time PCR system (Thermo Fisher Scientific) with the following protocols: (a) For the Sansure PCR kit, there was an initial 50°C, 30‐minutes step for reverse transcription followed by a 95°C, 1‐min cDNA pre‐denaturation step, then 45 cycles at 95°C for 15 seconds and 60°C for 30 seconds for denaturation, annealing (with fluorescence monitoring), and an elongation step, and finally a 25°C, 10‐s step for instrument cool down; (b) For the BioGerm PCR kit, there was an initial 50°C, 10‐minutes step for reverse transcription followed by a 95°C, 5‐min cDNA pre‐denaturation step, then 40 cycles at 95°C for 10 seconds, and 60°C for 40 seconds for denaturation, annealing (with fluorescence monitoring), and an elongation step. Quality control and assurance, including three internal positive controls and a negative control, were included in each run to identify the false‐negative and false‐positive results. Furthermore, three parallel tests were performed with three weakly positive samples, the test for each sample was conducted simultaneously, using two different PCR kits but the same amplification machine.

### 2.5 Analysis of the results

The test results were determined based on the cycle threshold (Ct) value. According to the instructions of the Sansure PCR kit, an s‐type amplification curve with Ct ≤ 40 represents a positive result, and Ct > 40 represents a negative result. For the BioGerm PCR kit, an s‐type amplification curve with Ct ≤ 35 indicates a positive result, and Ct > 38 indicates a negative result. If the amplification curve is between 35 and 38, the result should be re‐checked. Only if the results remain consistent can a result be treated as positive.

### 2.6 Statistical analyses

The data are presented qualitatively. The test results of patients with COVID‐19 and patients without COVID‐19 were collected and analyzed. To evaluate the detection efficiency of these two PCR kits and their diagnostic value, the sensitivity, specificity, positive predictive value (PPV), negative predictive value (NPV), kappa value, and their 95% confidence intervals(CI) were calculated. The in‐batch repeatability of different reagents was compared with the coefficient of variation (CV). All data analyses were conducted in SPSS version 16.0 (SPSS Inc, Chicago, USA).

## 3 RESULTS

Throat swab samples from 18 patients with COVID‐19 and 100 patients without COVID‐19 were successfully analyzed. The detailed Ct values of all samples are shown in Table S1. For the Sansure PCR kit, 3 of the 18 samples were false‐negative results, and for the BioGerm PCR kit, 1 of the 18 samples was a false‐negative result. No false‐positive results were detected in this test. The sensitivity, specificity, PPV, NPV, and kappa value of the Sansure PCR kit were 0.833, 1.000, 1.000, 0.971, and 0.894, respectively, and the sensitivity, specificity, PPV, NPV, and kappa value of the BioGerm PCR kit were 0.944, 1.000, 1.000, 0.990, and 0.966, respectively(Table 2). These results indicated that the detection efficacy of the BioGerm PCR kit for SARS‐CoV‐2 nucleic acid detection was relatively higher than that of the Sansure PCR kit.

**Table 2:** Diagnosis efficacy of Sansure and BioGerm test kits for SARS‐CoV‐2 nucleic acid detection

	COVID‐19 samples(n = 18)		None‐ COVID‐19 samples(n = 100)		Sensitivity (95%CI)	Specificity (95%CI)	PPV (95%CI)	NPV (95%CI)	Kappa (95%CI)
Test kits	Positive	Negative	Positive	Negative					
Sansure	15	3	0	100	0.833(0.577‐0.956)	1.000(0.954‐1.000)	1.000(0.747‐1.000)	0.971(0.911‐0.992)	0.894(0.726‐1.000)
BioGerm	17	1	0	100	0.944(0.706‐0.997)	1.000(0.954‐1.000)	1.000(0.771‐1.000)	0.990(0.938‐0.999)	0.966(0.880‐1.000)

For the three parallel tests, the results of the Sansure PCR kit showed one of the ORF1ab from sample 2 was not detected. As shown in Table 3, the CV value of the BioGerm PCR kit in all three samples was the smallest for both the ORF1ab and N gene, indicating that the reproducibility of in‐batch detections with the BioGerm PCR kit was better than that with the Sansure PCR kit.

**Table 3:** The parallel test result of Sansure and BioGerm test kits for SARS‐CoV‐2 nucleic acid detection

COVID‐19 samples	Sansure		BioGerm	
	Orf1ab	N	Orf1ab	N
Sample 1	33.94	23.54	32.06	28.29
Sample 1	31.95	20.87	30.03	27.84
Sample 1	28.79	21.71	28.44	27.81
CV(%)	8.23	6.19	6.01	0.96
Sample 2	33.51	26.13	33.82	31.47
Sample 2	(‐)	22.40	31.37	30.70
Sample 2	30.94	25.66	32.50	31.32
CV(%)	5.64	8.21	3.77	1.31
Sample 3	32.17	30.69	31.68	31.72
Sample 3	32.58	31.26	32.28	33.93
Sample 3	29.85	30.32	30.65	32.74
CV(%)	4.67	4.56	2.61	3.37

## 4 DISCUSSION

Herein, we compared two commercially available RT‐PCR kits for the detection of SARS‐CoV‐2 in clinical samples. These two kits had the same specificity and PPV for SARS‐CoV‐2 nucleic acid detection; however, the sensitivity, NPV, and kappa value of the BioGerm PCR kit were all higher than those of the Sansure PCR kit; for the parallel tests, the CV value of the BioGerm PCR kit in all three samples was also smaller and more stable than that of the Sansure PCR kit, suggesting that the detection efficacy of the BioGerm PCR kit for SARS‐CoV‐2 nucleic acid was better than that of the Sansure PCR kit.

COVID‐19 is an emergent public health hazard, and its outbreak has caused reagent manufacturers to develop and obtain approval for nucleic acid testing kits in a short time, which may have led to some defects in setting the performance parameters of the kits. Therefore, in‐house clinical validations upon implementation of novel RT‐PCR kits need to be conducted. Thus far, several studies have been devoted to this topic, but the majority of them assessed these products using different kits.^9^, ^10^, ^11^, ^12^ Only a study by Shen et al evaluated the same Sansure PCR kit that we evaluated,^10^ and they found sensitivity, specificity, PPV, NPV, and kappa values of 95.00%, 87.50%, 95.00%, 87.50%, and 0.825, respectively, for the Sansure PCR kit (Lot No. 2020003), which were quite differ from our results. However, the data are incomparable because the lot number of the PCR kit we used was different (2020003 in their study and 2020007 in our study).

In the present study, we found that the BioGerm PCR kit had better detection efficacy than the Sansure PCR kit. However, we performed our analysis using a small number of clinical samples, and only one lot of these two kits was used (Lot No. 2020 07 and 20200304A for the Sansure and BioGerm PCR kits, respectively). These kits do not necessarily represent the overall performance of the Sansure and BioGerm PCR kits, and additional and more extensive clinical validations should be conducted. In addition, most of the clinical samples we used in the present study had low viral loads (CT value > 30), and such samples have higher sensitivity requirements for the detection kit. If the minimum detection limit cannot reach the detection concentration, weakly positive samples might show a false‐negative result.^13^

In summary, we reported the detection performance of two different SARS‐CoV‐2 nucleic acid detection kits using clinical samples. While both the assays that were evaluated were highly specific, the BioGerm PCR kit was more sensitive than the Sansure PCR kit. These findings provide important information for the ongoing optimization of viral detection assays following the emergence of COVID‐19.

## AUTHOR CONTRIBUTIONS

YL drafted the manuscript; XL, LZ and WL participated in the sample collection, LL and SR carried out the qRT‐PCR, LL and HY participated in the design of the study and performed the statistical analysis.

## Supporting information

Table S1

## Data Availability

The datasets used and/or analyzed during the current study are available from the corresponding author on reasonable request.
