# Supplementary material for: Comparison of the diagnostic efficacy between two PCR test kits for SARS‐CoV‐2 nucleic acid detection
Source: J Clin Lab Anal. 2020 Sep 25;34(10):e23554. doi: 10.1002/jcla.23554 (PMC7536918; doi:10.1002/jcla.23554)
Supplement: Supplementary file 1 — Table S1 [file JCLA-34-e23554-s001.docx]

Table S1 The detailed Ct values of the COVID-19 samples using two different test kits.

| COVID-19 samples | Sansure | | BioGerm | |
| --- | --- | --- | --- | --- |
| Sample ID(n=18) | Orf1ab | N | Orf1ab | N |
| 1 | 30.09 | 29.79 | 32.49 | 30.88 |
| 2 | 32.02 | 29.40 | 33.75 | 32.84 |
| 3 | 37.29 | 31.19 | 34.31 | 34.42 |
| 4 | 32.17 | 30.69 | 31.68 | 31.72 |
| 5 | 32.58 | 31.26 | 32.28 | 33.93 |
| 6 | 38.92 | 33.44 | 34.31 | 34.74 |
| 7 | 29.85 | 30.32 | 30.65 | 29.95 |
| 8 | 28.30 | 28.68 | 33.25 | 34.99 |
| 9 | 27.17 | 25.86 | 28.92 | 32.07 |
| 10 | （—） | 26.63 | 32.19 | 30.53 |
| 11 | 34.92 | 31.89 | 33.89 | 36.95 |
| 12 | 33.94 | 23.54 | 32.06 | 28.29 |
| 13 | 31.95 | 20.87 | 30.03 | 27.84 |
| 14 | 29.79 | 27.66 | 29.99 | 28.42 |
| 15 | （—） | 28.84 | 31.68 | 31.11 |
| 16 | （—） | 29.13 | （—） | 30.68 |
| 17 | 34.95 | 22.78 | 31.55 | 30.05 |
| 18 | 28.79 | 21.71 | 28.44 | 27.81 |
